# Characterization and diagnostic evaluation of hypersensitivity to iodinated contrast media: A retrospective analysis^☆^

**DOI:** 10.1016/j.waojou.2026.101366

**Published:** 2026-03-19

**Authors:** Gabija Brusokienė, Kotryna Linauskienė, Kęstutis Černiauskas, Linas Griguola, Justina Rudytė, Rūta Vėbrienė, Anželika Chomičienė, Laura Malinauskienė

**Affiliations:** a Vilnius University, Faculty of Medicine, Institute of Clinical Medicine, Clinic of Chest Diseases, Immunology and Allergology, Vilnius, Lithuania; b Vilnius University Hospital Santaros Klinikos, Pulmonology and Allergology Department, Vilnius, Lithuania

**Keywords:** Contrast media, Drug hypersensitivity, Skin tests, Drug provocation test, Risk assessment

## Abstract

**Background:**

Hypersensitivity reactions to iodinated contrast media (ICM) pose a growing clinical challenge, especially for patients requiring imaging. Differentiation of immune-mediated hypersensitivity from non-immune adverse reactions is essential to avoid unnecessary ICM avoidance and ensure safe diagnostic imaging procedures.

**Methods:**

We conducted a retrospective analysis of 222 patients assessed for suspected ICM hypersensitivity at Vilnius University Hospital Santaros Clinics from 2017 to 2024. All patients underwent skin prick and intradermal testing. Selected individuals received intravenous provocation testing. Clinical characteristics, symptom profiles, and outcomes were assessed.

**Results:**

Sensitization to ICM was confirmed in 16.67% of patients, primarily via intradermal and provocation testing. Urticaria (OR = 3.93) and anaphylaxis (OR = 4.17) were significantly associated with confirmed sensitization, while isolated non-cutaneous symptoms and late-onset angioedema were not. Higher sensitization rates were observed in patients with oncologic (29.63%) and cardiovascular (20.69%) conditions, and renal pathology showed the lowest rate (2.94%). Among non-sensitized patients, 75 underwent ICM imaging without adverse reactions and were safely delabeled. Most sensitized individuals tolerated alternative ICM agents.

**Conclusions:**

A tiered allergological approach using skin and provocation testing supports accurate diagnosis and risk-stratified imaging strategies, including safer alternative ICM selection and selective delabeling. Findings support early referral, personalized contrast selection, and delabeling strategies in high-risk populations.

## Introduction

Iodinated contrast media (ICM) are indispensable in modern radiology, yet hypersensitivity reactions remain a clinically significant concern.^1^^,^^2^ Although the overall rate of adverse reactions with current non-ionic, low-osmolar ICM is low,^3^ increasing use has led to more hypersensitivity reactions.^4^ These reactions range from mild urticaria to life-threatening anaphylaxis.^5^, ^6^, ^7^, ^8^ Contemporary consensus recognizes two mechanistic categories of immediate reactions (IHRs): immune-mediated (including IgE-mediated) reactions, and non-IgE “infusion-type” reactions, driven by direct mast-cell activation and physicochemical properties (eg, MRGPRX2-mediated pathways).^9^

These mechanisms are clinically indistinguishable but have major implications for diagnostic sensitivity and management, since skin testing and provocation primarily detect IgE- or T-cell–mediated pathways and do not reliably reproduce non-IgE immediate reactions.^10^, ^11^, ^12^, ^13^, ^14^ Non-immediate hypersensitivity reactions (NIHRs), defined as onset >1 h after administration per EAACI recommendations, are typically T-cell–mediated and present predominantly with cutaneous eruptions, though severe cutaneous adverse reactions are rare.^10^, ^11^, ^12^, ^13^, ^14^

Despite available diagnostic tools — including skin prick testing (SPT), intradermal testing (IDT), and drug provocation testing (DPT) — their real-world use and predictive value vary.^4^ Many patients are inappropriately labelled as “iodine-allergic,” leading to unnecessary avoidance of essential contrast-enhanced imaging, delays in diagnosis, and increased healthcare burden. ^4^^,^^14^, ^15^, ^16^

Accurate risk stratification is therefore essential, particularly for high-utilization groups such as oncology and cardiovascular patients who frequently undergo contrast-enhanced procedures. 15, 16, 17

This study aimed to assess, in a real-world tertiary allergy center population, the diagnostic yield of a structured testing algorithm (SPT → IDT → selective intravenous DPT), identify clinical predictors of confirmed sensitization, and evaluate outcomes of re-exposure, including delabeling. Emphasis was placed on mechanism-based interpretation, recognizing that negative tests reduce — but do not eliminate — the risk of recurrent non-IgE immediate reactions.

## Methods

### Study population

This retrospective study included 222 patients referred to the allergology day center at Vilnius University Hospital Santaros Clinics between 2017 and 2024 due to suspected ICM hypersensitivity, or to clarify an existing ICM/“iodine” allergy label. Referrals comprised three groups: (1) patients with a prior reaction temporally related to ICM administration (n = 204); (2) patients with previous ICM exposure without adverse reactions but concern about future use (n = 14); and (3) patients without prior ICM exposure but labelled “iodine-allergic” or reporting reactions to iodine-containing products, which had led to avoidance of ICM-enhanced imaging (n = 4). The primary analysis of diagnostic yield and predictors was restricted to group (1), groups (2) and (3) are described to reflect the real-world referral population. Patients were included if they had undergone allergological evaluation, including skin testing and/or intravenous DPT with ICM. Immediate hypersensitivity reactions (IHRs) were defined as reactions with onset ≤1 h after ICM administration, and non-immediate hypersensitivity reactions (NIHRs) as reactions with onset >1 h, in accordance with current EAACI recommendations. Collected variables included age, gender, referral reason, reaction features (symptoms, immediate vs delayed, time since reaction, implicated ICM), comorbidities, prior non-ICM adverse drug reactions, inhalant allergen sensitization, and the need for further ICM-enhanced imaging.

### Allergy testing

Skin testing followed a standardized diagnostic protocol of our center. All patients underwent SPT (undiluted ICM) followed by IDT (1:10 dilution in normal saline) to iopromide, iohexol, iodixanol, and diatrizoate. Skin testing was performed on the volar surface of the forearm. IDT volumes were 0.02–0.03 ml per injection site. Normal saline served as the diluent and negative control, histamine was used as positive control for SPT. Immediate readings were taken at 15–20 min and again at ∼1 h for IDT. Delayed IDT readings at 24 and 48 h were obtained when NIHR was suspected. Test was considered positive if there was a wheal enlargement of ≥3 mm with surrounding erythema compared to the negative control, following EAACI/ENDA recommendations.^18^^,^^19^

Intravenous DPT was performed only when the clinically required or alternative ICM had negative skin tests or clinical suspicion remained high or documentation of a safe alternative was needed or non-allergic explanations were insufficient. The protocol used incremental intravenous dosing (1 ml → 10 ml → 20–30 m) at 30-min intervals to a cumulative 31–41 ml, under continuous monitoring with resuscitation available.

### Statistical analysis

All data was analyzed using SPSS v28.0, UpSetR package for co-occurrence visualizations, and Microsoft Excel for data handling/graphs. Descriptive statistics were used to summarize demographic and clinical characteristics. We summarized demographics and clinical data descriptively and assessed associations using chi-square tests, Spearman's ρ, and odds ratios (OR) with 95% confidence intervals (CI). Statistical significance was set at p < 0.05.

#### Ethics

This study was approved by the Vilnius Regional Bioethics Committee (approval No. 158200-16-847-355) (Lithuania) and conducted in accordance with ethical standards specified in the Declaration of Helsinki.

## Results

### Cohort and index reaction characteristics

Among 222 patients 178 (80.20%) were female and 44 (19.80%) male, with a mean age of 59.71 ± 14.09 years. Referral indications fell into three primary categories. Most patients (n = 204; 91.89%) had prior ICM reaction, A smaller group (n = 14; 6.31%) had prior ICM exposure without reaction but concern about future use. The remaining patients (n = 4; 1.80%) had never received ICM but were referred due to significant anxiety about potential allergic reactions. These individuals reported hypersensitivity to iodine-containing topical antiseptics (e.g., povidone-iodine), and in all cases, imaging with ICM had been declined or postponed due to these concerns. Referrals were made either at the patient's request or upon recommendation from treating physicians who suspected a possible iodine-related hypersensitivity. Although these cases lacked prior systemic ICM exposure, their inclusion was clinically justified, as unnecessary avoidance of contrast-based imaging posed a barrier to appropriate diagnostics.

Sensitization to inhalant allergens was identified in 23 patients (10.36%), 44 patients (19.82%) reported adverse drug reactions unrelated to ICM. 101 (45.5%) patients were referred due to the anticipated need for future radiological procedures involving ICM.

**Fig. 1:**
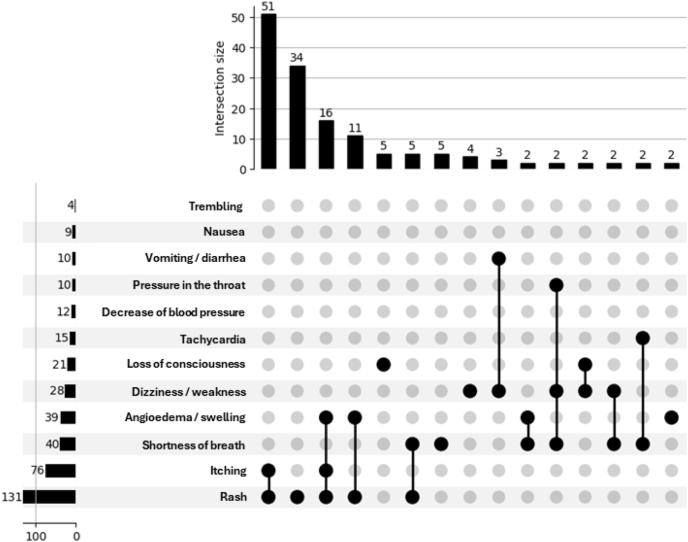
**Distribution of symptoms following ICM administration**. Only most frequently observed symptom combination presented

Culprit ICM was documented in 126/222 (56.8%) cases, most commonly Iopromide (n = 54), Iohexol (n = 36), Iodixanol (n = 23), and Diatrizoate (n = 13). Reported symptoms included rash (n = 131), itching (n = 76), dyspnea (n = 40), and angioedema/swelling (n = 39). The most frequently observed symptom combination was rash and itching (n = 51) (Fig. 1). Timing of the index reaction was available in 191/222 patients: IHRs occurred in 97 patients (50.79%), and NIHRs in 94 patients (49.21%). In 31/222 (14.0%) patients onset could not be reliably retrieved.

### Diagnostic work-up and confirmation of hypersensitivity

**Fig. 2:**
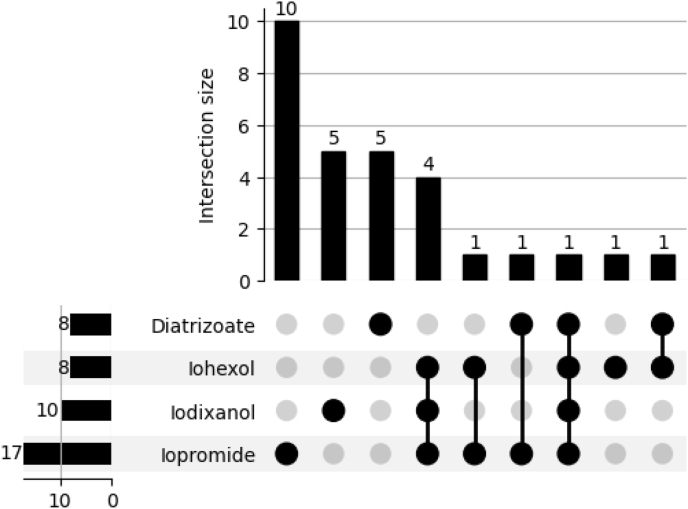
**Confirmed sensitization to iodinated contrast agents by intradermal testing with ICM**.

SPT was negative in all patients. IDT identified sensitization in 29 patients (13.06%), most frequently to Iopromide (n = 17). 24.32% of IDT-positive patients reacted to multiple ICM agents, suggesting a potential for cross-reactivity (Fig. 2).

**Fig. 3:**
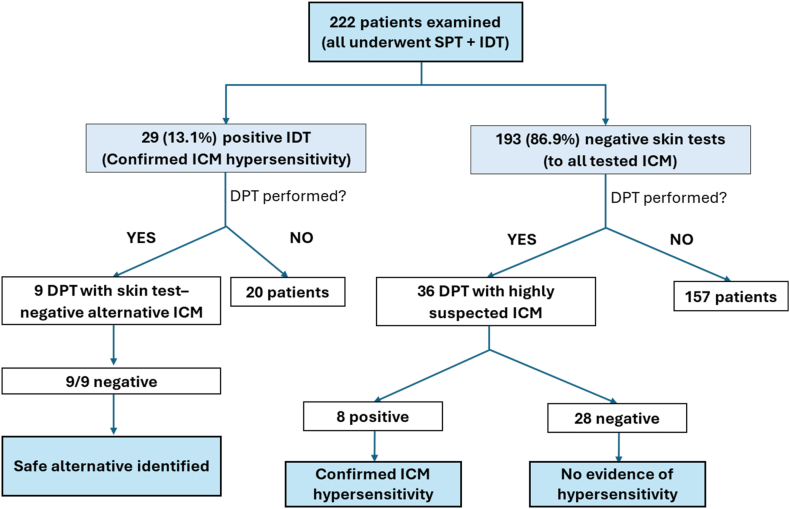
**Diagnostic work-up and outcomes in 222 patients evaluated for suspected or potential hypersensitivity to iodinated contrast media**. All patients underwent skin testing. A subset underwent intravenous drug provocation testing with skin test–negative agents. Confirmed hypersensitivity was based on positive intradermal testing and/or positive DPT. Non-sensitized patients with subsequent uneventful re-exposure were considered tolerant

DPT was performed in 45 patients, either due to a compelling clinical history or to confirm the safety of alternative contrast agents, always using skin test-negative agents. 9 DPTs were performed to confirm safe alternatives in IDT-positive patients (all negative) and 36 because of strong clinical suspicion despite negative skin tests. Eight of these 36 patients (3.60% of total cohort) reacted only on DPT despite negative SPT/IDT (iodixanol n = 3, iohexol n = 3, iopromide n = 1, and iohexol + iopromide n = 1). Overall, sensitization to at least one ICM was confirmed in 37 patients, corresponding to 18.1% (37/204) of patients with a prior ICM reaction (primary analysis population) and 16.7% (37/222) of the entire referred cohort. Sensitization rates did not differ by sex: (women 16.85% vs men 15.91%, p = 1.0). (Fig. 3). IHR were more frequently associated with positive skin tests (n = 19) than NIHR (n = 10). Conversely, delayed reactions were more commonly identified through intravenous provocation testing (n = 6) than immediate reactions (n = 2).

### Predictors of confirmed sensitization

**Fig. 4:**
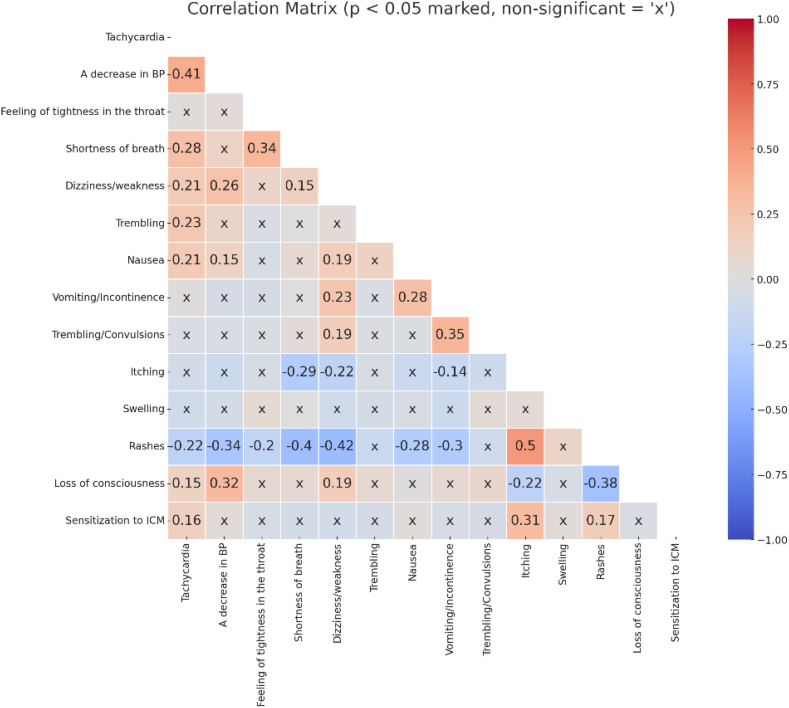
**Correlation between clinical symptoms and confirmed sensitization**. Only statistically significant correlations (p < 0.05) are shown with numerical values; non-significant correlations are marked with “x”. Positive correlations are shown in red, and negative in blue. The strength of the correlation is indicated by the colour intensity and value

An analysis of symptom occurrence in relation to ICM sensitization revealed distinct associations. Sensitization was moderately positively correlated with itching (ρ = 0.31, *p* < 0.05) and rash (ρ = 0.20, *p* < 0.05), and negatively correlated with tachycardia (ρ = −0.17, *p* < 0.05) (Fig. 4).

**Fig. 5:**
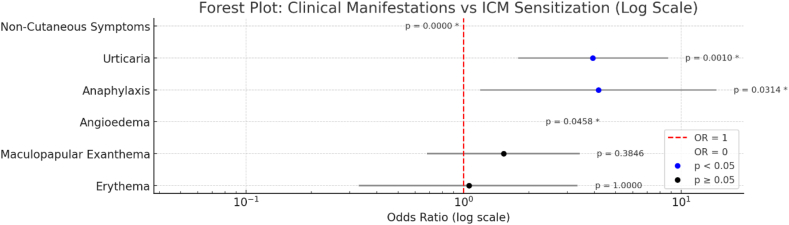
**Odds Ratios for Sensitization by Symptom Group**. Odds ratios (OR) with 95% confidence intervals (CI) are shown on a logarithmic scale for each clinical manifestation. Blue colors indicate statistically significant associations (p < 0.05), while black color represent non-significant results. Red “X” markers denote cases with an OR of 0. The vertical red dashed line represents the null value (OR = 1), indicating no association. P-values are displayed next to each point, with asterisks denoting statistical significance. The manifestation “Skin Blistering Reaction” was excluded from the analysis

In grouped phenotype analysis (Fig. 5; Table 1), anaphylaxis showed the strongest association with sensitization (n = 5/11; 45.46%; OR = 4.17, 95% CI: 1.19–14.54, *p* = 0.0314), followed by urticaria including cases accompanied by angioedema (15/40, 19.05%; OR = 3.93, 95% CI: 1.78–8.7, *p* = 0.001). Maculopapular exanthema (11/40; 23.40%; OR = 1.53, 95% CI: 0.68–3.42, *p* = 0.3846) and erythema, including both typical and pruritic forms (4/21; 19.05; OR = 1.06, 95% CI: 0.33–3.35, *p* = 1.0000) were not significantly associated. No sensitization was observed with isolated late-onset angioedema (0/17; OR = 0.00, 95% CI: 0.01–1.99, *p* = 0.0458) or isolated non-cutaneous symptoms (0/54; OR = 0.00, 95% CI: 0.00–0.45, *p* < 0.0001).

**Table 1:** Odds Ratio Between Clinical Manifestations and Confirmed ICM sensitization.

Manifestation	OR	95% CI lower	95% CI upper	p-value	Sensitized (n)	Total in Group
Anaphylaxis	4.17	1.19	14.54	0.0314	5	11
Late-onset angioedema	0	0.01	1.99	0.0458	0	17
Erythema	1.06	0.33	3.35	1	4	21
Maculopapular exanthema	1.53	0.68	3.42	0.3846	11	47
Non-cutaneous symptoms	0	0	0.45	0	0	54
Urticaria	3.93	1.78	8.7	0.001	15	40

∗For some patients, the anamnesis was limited to differentiate into clinical presentation groups

Sensitization rates differed by the clinical indication for imaging. The highest rate was found in patients with oncological diseases (16/54, 29.63%), followed by cardiovascular disease (6/29, 20.69%) and “other specific cases” (2/10, 20.00%). In contrast, patients with kidney disease had the lowest rate of sensitization (1/34, 2.94%). Overall differences between groups were statistically significant (χ^2^ = 12.52, *p* = 0.0284).

### Outcomes and follow-up

**Fig. 6:**
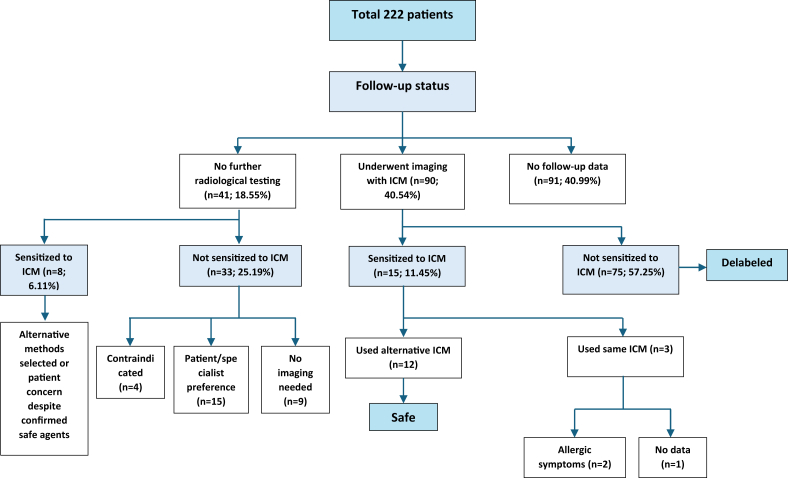
**Clinical pathways following allergy evaluation for ICM**.

Post-testing outcomes assessed subsequent imaging safety and use of ICM. Overall, 18.55% of patients underwent no further radiological testing, 40.54% underwent imaging with ICM, and follow-up was unavailable for 40.99%. Among non-sensitized patients with follow-up, 75 (33.78% of the total group and 57.25% of those with follow-up data) safely underwent ICM-enhanced imaging and were delabeled. This allowed them to access needed imaging procedures without unnecessary restrictions. However, 33 non-sensitized patients (25.19% of those with follow-up data) did not undergo ICM imaging. 15 patients avoided further imaging altogether, although sensitization was not confirmed, imaging was conducted without ICM or MRI was preferred (due to patient refusal or physician choice). 9 had no clinical need for further ICM imaging, and 4 patients were excluded from ICM imaging due to impaired renal function. Among sensitized patients with follow-up, 8 (6.11% of those with follow-up data) did not undergo imaging with ICM (alternative modalities selected or persistent patient concerns despite identifying safe alternatives). Of the 15 sensitized patients who underwent imaging procedures, 12 received alternative ICM agents identified as safe during allergy testing and tolerated the procedures without allergic symptoms. However, 3 patients were re-exposed to the culprit ICM, with recurrent reactions in 2 and no follow-up available for 1 (Fig. 6).

## Discussion

### Mechanistic interpretation and diagnostic approach

Using a tiered strategy (SPT, IDT, and selective intravenous DPT), we confirmed ICM hypersensitivity in 16.7% of all referrals (18.1% among those with a prior reaction). As in prior studies, SPT provided no diagnostic yield, whereas IDT (13.06%) and DPT (3.60%) contributed complementary value.^4^ The absence of positive SPT is consistent with reports of limited utility even in anaphylaxis presentations,^4^ while IDT appears essential and is repeatedly shown to be more sensitive, particularly in systemic or severe reactions.^4^^,^^20^^,^^21^ DPT identified additional sensitized patients undetected by skin testing and remains the gold standard for confirming hypersensitivity and establishing safe alternatives.^13^^,^^20^^,^^22^^,^^23^ We used a reduced-dose DPT protocol (31–41 ml), as a deliberate risk–benefit choice. While ENDA/EAACI recommend titrating to the maximum single therapeutic or even full cumulative dose, we adopted a conservative cumulative volume of 31–41 ml to mitigate nephrotoxic and pseudoallergic risks from renally excreted ICM while still yielding clinically meaningful data.^19^ Evidence supports such reduced-dose challenges can be informative: low-dose regimens (as low as 17 ml) show comparable diagnostic performance with no excess breakthrough reactions versus full-dose pathways^24^^,^^25^ and even 10 ml challenges have identified immediate-type reactions in selected reports.^26^ Importantly, negative DPT results should be interpreted as risk-reducing rather than risk-eliminating, particularly for dose or infusion rate dependent non-IgE immediate reactions. In parallel, the heterogeneity of ICM hypersensitivity mechanisms provides a biological rationale for imperfect correlation between symptoms and test outcomes: immediate reactions may involve IgE-dependent and non-IgE pathways, while non-immediate reactions are often T-cell mediated.^23^

### Clinical implications

A focused clinical phenotype may help predict which patients were most likely to have confirmed sensitization and therefore may support pragmatic triage for allergy testing. Cutaneous presentations remain central in ICM hypersensitivity syndromes,^7^^,^^21^^,^^27^ and in our cohort the highest-yield phenotypes were anaphylaxis-like presentations (OR = 4.17), and urticaria (OR = 3.93), consistent with prior observations that greater reaction severity and urticarial phenotypes are associated with higher rates of confirmed allergy.^4^^,^^7^^,^^21^^,^^23^^,^^27^^,^^28^ In contrast, isolated non-cutaneous complaints (e.g., dizziness, nausea, chest discomfort) rarely resulted in confirmed sensitization in our cohort, suggesting that many represent non-allergic adverse reactions or procedure-related physiology rather than immune-mediated hypersensitivity.^23^^,^^29^ Similarly, the negative association observed for tachycardia is compatible with non-immune mechanisms (e.g., direct mast-cell or complement activation), vasovagal responses, or situational factors that are not reliably captured by current diagnostic tools.^7^^,^^21^^,^^27^^,^^28^ Late-onset angioedema was also not linked to confirmed sensitization, which may reflect heterogeneous or poorly defined pathways not captured by current diagnostics.^7^^,^^23^ These data support symptom-guided approach: prioritize full evaluation for patients with cutaneous and/or multisystem features, especially anaphylaxis or urticaria, while isolated tachycardia or other non-cutaneous complaints may justify a more individualized approach rather than routine IDT/DPT without additional context.^7^^,^^23^^,^^28^

Clinical context further supports risk-stratified decision-making. Confirmed ICM sensitization was highest in oncology (29.63%), echoing reports of disproportionate ICM hypersensitivity in this group despite unclear mechanisms.^30^ While cancer or its treatments are not established independent risk factors, repeated exposures through frequent contrast-enhanced imaging may plausibly contribute.^22^^,^^30^^,^^31^ A multicenter study reported a higher incidence of IHR (2.1%) in cancer patients compared to 1.1% in non-cancer patients^30^ and prior work has noted that patients with hypersensitivity reactions to ICM were more likely to report a history of cancer (56.6%) compared to those reacting to gadolinium-based contrast media (GBCM).^32^ Emerging evidence also implicates cancer immunotherapy as a potential co-factor for hypersensitivity or anaphylaxis following imaging.^7^^,^^33^^,^^34^ Cardiovascular disease was also associated with elevated sensitization (20.69%), consistent with multivariate analyses identifying it as a risk factor,^22^^,^^35^^,^^36^ and with reports of cardiovascular disease being more common among ICM reactors (eg, 27.7%) than among those reacting to other contrast agents.^7^^,^^33^^,^^34^ By contrast, renal disease showed a very low sensitization rate (2.94%), aligning with literature in which renal status is not a consistent predictor.^7^ Large analyses more often implicate drug-allergy history, asthma, iodine content, or outpatient status, rather than renal impairment per se.^5^ Collectively, these patterns support risk-stratified pathways: proactive allergy evaluation may be particularly valuable in oncology and cardiovascular patients who both show higher confirmed sensitization and frequently require repeated imaging, whereas routine work-up in renal patients may be less efficient without additional risk factors. This approach aligns with proposals for risk classification systems and structured pre-procedural risk assessment in high-throughput imaging settings.^13^^,^^20^^,^^22^^,^^23^

Among sensitized patients, 24.32% reacted to multiple ICMs, indicating possible cross-reactivity or individual polyvalent sensitivity. Although lower than reports up to 73.3% across immediate and non-immediate reactions,^12^^,^^37^, ^38^, ^39^, ^40^, ^41^ this pattern aligns with systematic polyvalent reactivity may be more common than simple structure-based class cross-reactivity and supports individualized selection of alternatives guided by testing.^42^^,^^43^ Variable provocation outcomes across alternative agents in prior studies further reinforce this approach.^8^^,^^27^^,^^30^^,^^43^ From a practical standpoint, structured evaluation enables both safe agent selection and delabeling, while recognizing that negative testing cannot fully exclude dose or infusion rate dependent non-IgE immediate reactions.^9^ Our study also highlights the clinical value of allergy testing and the opportunity for safe delabeling in patients who are not sensitized. In practice, management after evaluation relies primarily on selection of an alternative ICM with negative testing, which is the preferred strategy to reduce recurrence risk. Premedication was used selectively in higher-risk situations as an adjunct to contrast substitution and careful monitoring, rather than as a replacement for allergy-guided agent choice. Therefore, “delabeling” in our cohort reflects a risk-stratified decision supported by negative evaluation and uneventful re-exposure, not proof of absolute future tolerance. In our cohort, 75 patients with negative results (57.25% of those with follow-up data) were delabeled after uneventful re-exposure, consistent with reports that negative skin tests — particularly when complemented by DPT — substantially reduce the likelihood of IgE or T-cell mediated ICM hypersensitivity and help identify safer alternatives 20, 21, 22, 23^,^^32^^,^^43^, ^44^, ^45^. Follow-up data for over half of our cohort (59.01%) and the occurrence of two breakthrough reactions after non-adherence to test-guided recommendations underscore the real-world importance of implementing results in subsequent imaging decisions.^46^

## Limitations

This study has several limitations. Its retrospective design may introduce selection or recall bias. Our cohort reflects all patients referred for assessment of suspected or potential ICM hypersensitivity, including a small subgroup without a prior ICM reaction but with anxiety or an “iodine allergy” label. Inclusion of these low-risk referrals may slightly dilute the overall proportion of confirmed hypersensitivity when calculated across the entire cohort. Follow-up data was unavailable for 41% of patients, limiting long-term outcome assessment. Additionally, as a single-centre study, generalizability may be constrained. In addition, intravenous DPT was performed only in a selected subgroup, and not all DPT-negative patients subsequently underwent contrast-enhanced imaging. Therefore, the true diagnostic sensitivity and real-world safety of our DPT protocol cannot be fully determined. Finally, we did not perform multivariable modelling. Associations between clinical characteristics and sensitization are therefore unadjusted and should be interpreted as exploratory.

## Future research

Future studies should explore the long-term immunologic mechanisms of ICM sensitization, particularly in oncologic and cardiovascular populations. Prospective, multicenter studies are needed to validate risk stratification models and optimize testing algorithms. Additionally, real-world trials assessing health system impact - e.g., reduction in diagnostic delays or improved imaging access - could further justify early allergy referral pathways.

## Conclusions

This retrospective study supports the clinical value of a structured diagnostic algorithm for evaluating suspected hypersensitivity to iodinated contrast media. Confirmed sensitization was found in 16.67% of referred patients, with intradermal and provocation testing proving more diagnostically valuable than skin prick testing. Urticaria and anaphylaxis-like reactions were strong clinical predictors of sensitization, whereas isolated non-cutaneous symptoms were not. Sensitization appeared more frequent among oncology and cardiovascular patients, although prior exposure burden could not be quantified in this dataset. Notably, most non-sensitized patients were successfully delabeled and safely underwent imaging, while sensitized patients largely tolerated alternative, skin-test-negative ICM. Overall, these findings favor a risk-stratified, test-guided approach: negative results should be interpreted as lowering the likelihood of immune-mediated hypersensitivity and helping select safer alternatives, rather than definitively excluding non-IgE immediate reactions or guaranteeing universal future tolerance.

## Abbreviations

ADR: Adverse Drug Reaction
BAT: Basophil Activation Test
CI: Confidence Interval
DPT: Drug Provocation Test
DRESS: Drug Reaction with Eosinophilia and Systemic Symptoms
EAACI: European Academy of Allergy and Clinical Immunology
ENDA: European Network on Drug Allergy
GBCM: Gadolinium-Based Contrast Media
ICM: Iodinated Contrast Media
IDT: Intradermal Test
IHR: Immediate Hypersensitivity Reaction
LTT: Lymphocyte Transformation Test
MRI: Magnetic Resonance Imaging
MPE: Maculopapular Exanthema
NIHR: Non-Immediate Hypersensitivity Reaction(s)
OR: Odds Ratio
SD: Standard Deviation
SJS: Stevens–Johnson Syndrome
SPSS: Statistical Package for the Social Sciences
SPT: Skin Prick Test
TEN: Toxic Epidermal Necrolysis

## Author contributions

All authors have read and approved the manuscript and significantly contributed to this paper. Conceptualization – KL, LM, AC. Data curation – GB, KČ, LG, JR, RV, KL, AC LM; Data analysis - GB; Investigation GB, KČ;, LG, JR, RV, KL, AC LM; Methodology - KL, LM, AC, KČ. Supervision - KL, LM, AC. Visualization - GB; Writing - original draft - GB; Writing - review & editing - GB, KČ;, LG, JR, RV, KL, AC LM.

## Ethics statement

The study was approved Vilnius Regional Bioethics Committee (No. 158200-16-847-355) before the review of clinical records.

## Data availability statement

The data related to our study and materials will be available upon request by contacting the correspondence author (Gabija Brusokienė, gabija.biliut@gmail.com).

## Disclosure of the use of generative AI and AI-assisted technologies

Nothing to disclose.

## Funding/financial support

No specific funding was received for this work.

## Conflict of interest

The authors report no competing interests.
